# Lens autophagy protein ATG16L1: a potential target for cataract treatment

**DOI:** 10.7150/thno.93864

**Published:** 2024-07-01

**Authors:** Yilei Cui, Xiaoning Yu, Jing Bao, Xiyuan Ping, Silu Shi, Yuxin Huang, Qichuan Yin, Hao Yang, Ruoqi Chen, Ke Yao, Xiangjun Chen, Xingchao Shentu

**Affiliations:** 1 Eye Center, The Second Affiliated Hospital, School of Medicine, Zhejiang University, Zhejiang Provincial Key Laboratory of Ophthalmology, Zhejiang Provincial Clinical Research Center for Eye Diseases, Zhejiang Provincial Engineering Institute on Eye Diseases, Hangzhou310009, China.; 2 The Institute of Translational Medicine, Zhejiang University, Hangzhou310020, China.

**Keywords:** ATG16L1, autophagy, cataract, ubiquitination, E3 ligase

## Abstract

**Rationale:** Cataract is the leading cause of blindness and low vision worldwide, yet its pathological mechanism is not fully understood. Although macroautophagy/autophagy is recognized as essential for lens homeostasis and has shown potential in alleviating cataracts, its precise mechanism remains unclear. Uncovering the molecular details of autophagy in the lens could provide targeted therapeutic interventions alongside surgery.

**Methods:** We monitored autophagic activities in the lens and identified the key autophagy protein ATG16L1 by immunofluorescence staining, Western blotting, and transmission electron microscopy. The regulatory mechanism of ATG16L1 ubiquitination was analyzed by co-immunoprecipitation and Western blotting. We used the crystal structure of E3 ligase gigaxonin and conducted the docking screening of a chemical library. The effect of the identified compound riboflavin was tested *in vitro* in cells and *in vivo* animal models.

**Results:** We used HLE cells and connexin 50 (cx50)-deficient cataract zebrafish model and confirmed that ATG16L1 was crucial for lens autophagy. Stabilizing ATG16L1 by attenuating its ubiquitination-dependent degradation could promote autophagy activity and relieve cataract phenotype in *cx50*-deficient zebrafish. Mechanistically, the interaction between E3 ligase gigaxonin and ATG16L1 was weakened during this process. Leveraging these mechanisms, we identified riboflavin, an E3 ubiquitin ligase-targeting drug, which suppressed ATG16L1 ubiquitination, promoted autophagy, and ultimately alleviated the cataract phenotype in autophagy-related models.

**Conclusions:** Our study identified an unrecognized mechanism of cataractogenesis involving ATG16L1 ubiquitination in autophagy regulation, offering new insights for treating cataracts.

## Introduction

Cataracts result from the opacification of the lens and are the leading cause of blindness and low vision globally 1. To date, the only established treatment is cataract surgery, which might cause a series of complications, such as posterior capsule opacification and endophthalmitis 2. Thus, there is a pressing need to elucidate underlying molecular pathogenesis and identify effective targeted therapies.

Autophagy is an evolutionarily conserved self-degradation process crucial in maintaining intracellular homeostasis 3, 4. In the lens, constitutively activated autophagy is observed throughout the differentiation of lens epithelial and fiber cells involved in organelle degradation and maintaining transparency 5, 6. Mutations in autophagy-related genes, such as FYCO1, EPG5, and CHMP4B, have been reported in congenital cataract patients 7-9. Autophagy defects in cataract-associated genes have also been confirmed in transgenic mouse models 10-12. For instance, mutations in TDRD7, a gene responsible for congenital cataracts, disrupt autophagosome fusion with lysosomes, resulting in a failure to remove organelles in lens fiber cells 13. Autophagy disturbance was also reported in age-related cataracts, with a prevalence of 54.38% in patients above age 60 14. These findings suggest the intricate role of autophagy in maintaining lens transparency and identify cataracts as an autophagy defect-related disease. However, the complex regulatory network of autophagy and its aberrancy, causing lens opacity, remains unclear.

More than 30 evolutionarily conserved autophagy-related genes (ATGs) are believed to be involved in the regulatory network, with ATG16L1 being a key determinant of autophagosome elongation 15, 16. It interacts with the ATG5-ATG12 conjugate to assemble the E3-like complex and facilitates LC3 lipidation for membrane biogenesis in higher eukaryotes 17, 18. Although its autophagic function has been extensively studied, little is known about the mechanisms regulating the ATG16L1 protein.

A recent study confirmed that ATG16L1 turnover is a ubiquitin-dependent process in COS 7 cells 19. In this study, we demonstrated that ubiquitination of ATG16L1 is crucial for regulating the autophagy pathway in the lens. We found that stabilizing ATG16L1 by attenuating its ubiquitination-dependent degradation could promote autophagy and alleviate the cataract phenotype in connexin 50 (*cx50*)-deficient zebrafish 4, a typical cataract model with a defective autophagy process. Mechanistically, this process weakens the interaction between E3 ubiquitin ligase gigaxionin and ATG16L1. E3 ubiquitin ligase-targeting drugs, specifically riboflavin, were effective in reducing ATG16L1 ubiquitination and activating autophagy, alleviating the cataract phenotype in zebrafish with *cx50* defect. Furthermore, we verified this mechanism in two other autophagy-related cataract models, including one induced by oxidative stress. Collectively, our results provide a promising strategy for a potential non-surgical cataract treatment.

## Materials and Methods

### Animals

AB WT and *cx50*-deficient zebrafish (Ensembl ID: ENSDARG00000015076), obtained from China Zebrafish Resource Center, were used in this study. Zebrafish (no defined gender until 60 days post-fertilization) were maintained in a circulating water system at 28.5 °C with a daily cycle of 14 hours of light/10 hours of dark. Embryos were collected and maintained in E3 media with methylene blue in a thermostatic incubator.

Our previous study identified that *cx50* deficiency in zebrafish results in defective autophagy, causing severe defects in organelle degradation and ultimately leading to cataract formation 20. Therefore, the *cx50*-deficient zebrafish was used as a cataract model with an autophagy defect in the present study.

In the rescue experiment, 80 µM riboflavin or DMSO was added to zebrafish at 12 hours post-fertilization (hpf), with media replacement every 24 hours until 72 hpf. For microinjection, 3-4 nl of ATG16L1 plasmid (50 ng/µl) was injected into fertilized eggs, and zebrafish were collected at 72 hpf.

For the H_2_O_2_-induced cataract zebrafish model, 2 µl of 3% H_2_O_2_ was injected into the lens anterior chamber of zebrafish using 32G needles as in previous studies 21. Control fish were injected with 0.5 µl of PBS. The culture medium of zebrafish was supplemented with or without 80 µM riboflavin-loaded liposomes for 24 hours and then placed in a 10 cm dish for further observation and photography.

Six-week-old Sprague-Dawley rats (male, 200 g, Shanghai SLAC Laboratory Animal Co., Ltd.) were used for the hypothermic cataract rat model. The lenses were obtained from post-mortem rats and incubated at 4 °C in Hibernate™-A Medium (A1247501; Thermo Fisher Scientific, Waltham, MA, USA) containing 10% fetal bovine serum (FBS) (AusgeneX, Brisbane, Australia) and 1% penicillin-streptomycin for 24 h. Subsequently, the lenses were washed 3 times with PBS and incubated in DMEM (10-090-CV; Corning, New York, USA) supplemented with 10% FBS with or without 80 µM riboflavin for 24 h. Following rewarming treatment, the rat lenses were placed in an Extracellular Solution (C0216; Beyotime Biotechnology, Shanghai, China) for further observation and photography.

All animal experiments in this study were conducted in accordance with the ARRIVE guidelines and were approved by Zhejiang University Animal Care and Use Committee protocols (No. 2019-069).

### Cell culture and transfections

HLE cells (SRA01-04) were obtained from the RIKEN Cell Bank (RCB1591), and HEK293T cells were acquired from ATCC (CRL-3216). The cells were cultured in DMEM (10-090-CV; Corning) containing 10% FBS. For RNA interference, the siRNA was transfected using Lipofectamine 3000 (L3000015; Invitrogen, Carlsbad, CA, USA) for 48 h, according to the manufacturer's instructions. Subsequently, RNAi efficiency was measured by Q-PCR and Western blotting. siRNAs used were: CX50 siRNA#1: GCCAGGTCAGACGATCTAA. CX50 siRNA#2: TGTCCCTATTCCTCAACGT. For gene overexpression, the cells were transiently transfected with the indicated plasmids using Lipofectamine 3000 for 24 h. Other reagents used were as follows: chloroquine (CQ, HY-17589A), 30 µM, 6 h; MG132 (HY-13259), 10 µM, 6 h; riboflavin (HY-B0456), 80 µM, 24 h were purchased from MedChemexpress, New Jersey, USA. Riboflavin-loaded liposomes, 80 µM, 24 h were acquired from AVT Pharmaceutical Technology.

### Immunoprecipitation (IP) and Western blotting

For IP, the cells were lysed in RIPA buffer (P0013; Beyotime Biotechnology) with 1% PMSF and then centrifuged at 12000 g for 30 min. The resultant supernatant was mixed with the indicated antibodies at 4 °C for 4 h, followed by adding A/G magnetic beads (B23202; Bimake, Houston, Texas, USA) at 4 °C overnight. After washing with RIPA lysis buffer 3 times, the beads were resuspended in 50 μl of 2× loading buffer and subjected to Western blotting, as described previously 22. Briefly, equal amounts of samples were separated by SDS-PAGE, transferred to PVDF membranes, and incubated with appropriate antibodies. The following antibodies were used: anti-ATG16L1 (PM040; MBL, Japan), anti-LC3 (3868; Cell Signaling Technology, Danvers, MA, USA), anti-P62 ( ab56416; Abcam, Cambridge, UK), anti-CX50 (33-4300; Invitrogen, Carlsbad, CA, USA), anti-GAPDH (60004-1-Ig; Proteintech, Chicago, Illinois, USA), anti-ATG12 (2011; Cell Signaling Technology), anti-ATG5 (12994; Cell Signaling Technology), anti-Flag (F3165; MilliporeSigma, Saint Louis, Missouri, USA), anti-HA (ab9110; Abcam), anti-Ubiquitin (PM040; MBL), and anti-MYC (2276; Cell Signaling Technology). The blots were analyzed using an image analysis system.

### RNA extraction and real-time RT-PCR

Total RNA extraction and real-time RT-PCR were conducted as described previously 22. The primers used were as follows:

ATG16L1-F: CAGGCACGAGATAAGTCCCG, ATG16L1-R: ACTCCCCACGTTTCTTGTGT; ATG5-F: AAAGATGTGCTTCGAGATGTGT, ATG5-R: CACTTTGTCAGTTACCAACGTCA; ATG12-F: TAGAGCGAACACGAACCATCC, ATG12-R: CACTGCCAAAACACTCATAGAGA.

### Immunofluorescence

The HLE cells or zebrafish were fixed with 4% paraformaldehyde (P1110, Solarbio) for 15 min at room temperature and then blocked in PBS containing 10% goat serum albumin and 0.4% Triton X-100 (P0096, Beyotime Biotechnology) for 1 h. Then, the cells were incubated with the corresponding primary, secondary antibodies and DAPI (C0065, Solarbio), as indicated. Confocal images were captured using a Nikon A1 confocal microscope.

### Transmission electron microscopy (TEM)

TEM was performed as described previously 22. The cells and zebrafish eyes were fixed with 2.5% glutaraldehyde overnight at 4 °C and postfixed with 1% OsO4(02595-BA; SPI Chem) followed by dehydration through graded ethanol and absolute acetone. The tissues were then embedded in Spurr resin, sectioned to a thickness of 65 nm, and then stained with 2% uranyl acetate (02624-AB; SPI Chem) and alkaline lead citrate. TEM images were captured with a Hitachi Model H-7650 TEM.

### Computational Screening

The data file for the structure of the human gigaxonin protein was downloaded from the AlphaFold database. All heterogeneous atoms were removed, followed by molecular docking. High-throughput virtual screening was performed by MCE Co., Ltd. (Shanghai, China) under HTVS, SP, and XP modes. The compounds were selected based on scoring values, conformation and structural diversity. Protein-ligand interactions were obtained by PyMOL version 1.7.4.5.

### Cellular thermal shift assay (CETSA)

HLE cells were seeded in 10 cm dishes and incubated with 80 µM riboflavin or DMSO for 24 h. RIPA lysis buffer was added, and the respective lysates were divided into PCR tubes (100 μL). The lysates were individually heated at different temperatures (55, 60, 65, 70, 75, 80, and 85 °C) for 5 mins using a thermal cycler, then cooled for 3 mins at room temperature. The heated lysates were centrifuged at 12,000 g for 15 mins at 4 °C. The supernatants were transferred to new Eppendorf tubes for subsequent analysis by Western blotting.

### Statistical analyses

The data were analyzed by GraphPad Prism 8.0 software and shown as the mean ± Standard Deviation (SD) of three independent experiments. The normality of the datasets was assessed using the Shapiro-Wilk normality test. The homogeneity of variances was examined using Levene's test. If the data were normally distributed, Student's t-test was used to compare the two groups. If the data were non-normally distributed, the Mann-Whitney U test was employed to compare the two groups. A value of *p < 0.05 was considered significant.

## Results

### ATG16L1 is crucial for lens autophagy induced by CX50 *in vitro* and *in vivo*

**Figure 1:**
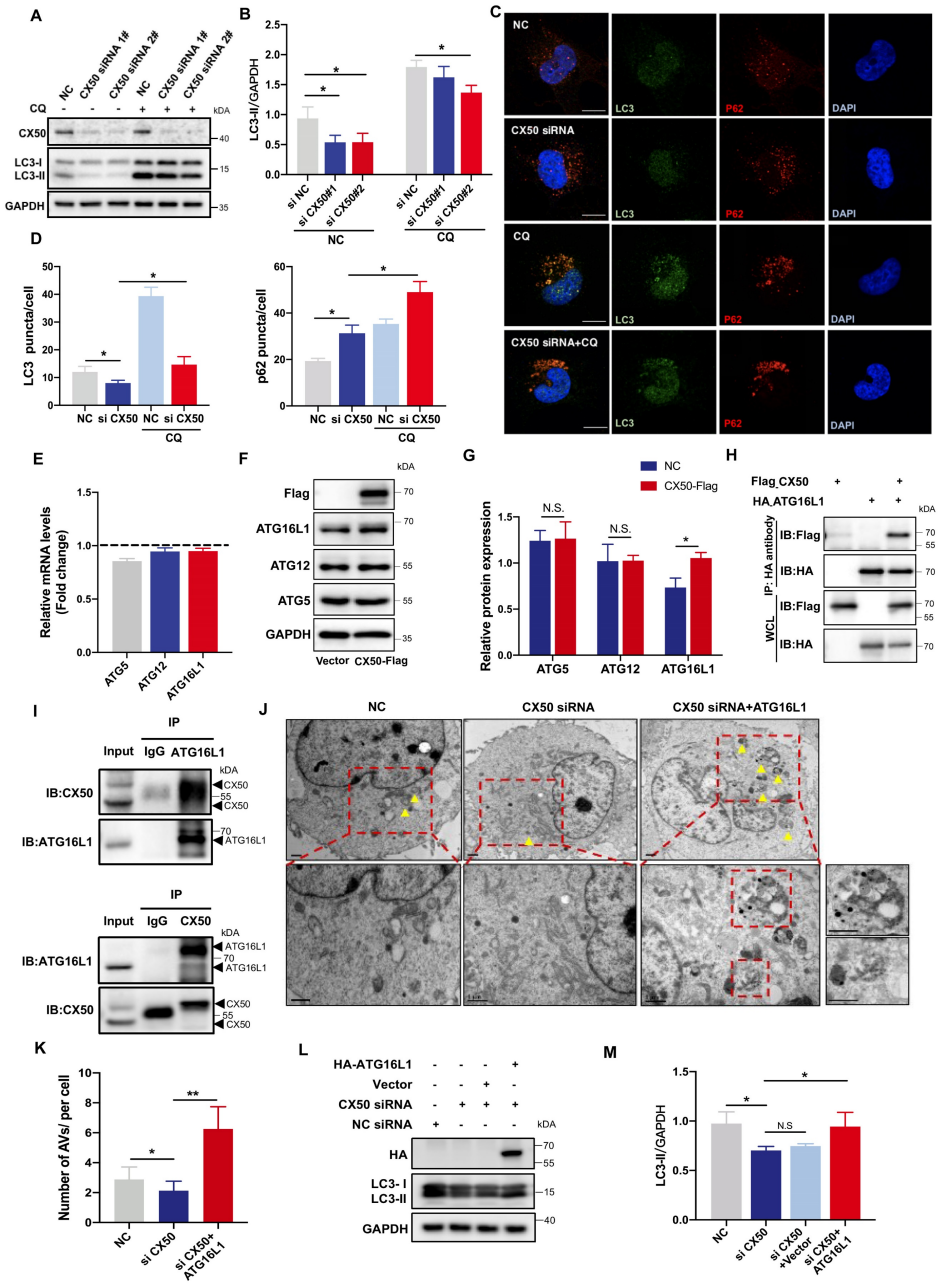
** ATG16L1 is crucial for lens autophagy induced by CX50.** (A) Western blot analysis of LC3-I/II and CX50 in NC or CX50-knockdown HLE cells treated with or without 30 µM CQ for 6 h. (B) Quantification of LC3 levels, n = 3. Mann-Whitney test. (C) Representative images of LC3 and p62 puncta in NC and CX50-knockdown HLE cells treated with or without CQ (n = 3, > 30 cells per experiment), scale bar: 10 µm. (D) Quantification of LC3 and p62 puncta per cell, n = 3, > 30 cells per experiment (Mann-Whitney test). (E-G) mRNA(E) and protein levels (F and G) of ATG5, ATG12, and ATG16L1 in CX50 over-expressing HLE cells. (H) Immunoblot of Co-IP from Flag-CX50 with HA-ATG16L1 in HEK293T cells. WCL: whole cell lysis. (I) IP analysis of the interaction between CX50 and ATG16L1 in HLE cells. (J) Electron micrographs of HLE cells and (K) quantification of the AVs per cell. Yellow arrows indicate autophagic vacuoles (AVs). ≥ 8 cells per experiment (Student's t-test analysis), scale bar: 1 µm. (L) Western blot analysis of LC3-II and HA in NC or CX50-knockdown HLE cells transfected with vector or ATG16L1. (M) Quantification analysis of LC3 levels, n = 3. Mean ± SD, *p < 0.05, **p < 0.01. N.S. not significant (Mann-Whitney test).

Autophagy plays a vital role in organelle degradation and homeostasis maintenance in the lens. We investigated the effect of cataract-associated gene CX50 on dynamic autophagic flux and assessed autophagosome formation in human lens epithelial cells (HLE cells) using two different CX50 siRNAs with the lysosomal inhibitor CQ. The expression level of MAP1LC3B/LC3B, a well-characterized autophagosome marker, was decreased in the CX50-knockdown cells with or without CQ (Figure 1A-B). Consistent with this expression pattern, we observed significantly decreased LC3 puncta and SQSTM1/p62 puncta accumulation with or without CQ, suggesting disrupted autophagic flux in HLE cells (Figures 1C-D). These results implied that CX50 deficiency leads to defective autophagy in the lens.

Based on our previous study 20, we speculated that CX50 might affect the early phase of autophagosome biogenesis. We examined the mRNA levels of ATG12, ATG5, and ATG16L1, three core autophagy proteins essential for early autophagosome formation, but found no significant changes in CX50-overexpressing HLE cells (Figure 1E). However, analysis of their protein levels showed elevated expression of ATG16L1 in CX50-overexpressing HLE cells (Figure 1F-G). In addition, co-immunoprecipitation (Co-IP) assays confirmed the interaction between Flag-tagged CX50 and HA-tagged ATG16L1 in HEK293T cells (Figure 1H). The endogenous CX50-ATG16L1 complex was also examined in HLE cells, confirming their direct association (Figure 1I). More importantly, transmission electron microscopy (TEM) showed an accumulation of enlarged double-membrane structures in the CX50 siRNA + ATG16L1 group, suggesting that ATG16L1 supplementation rescued the suppressive effect of CX50-knockdown on autophagy (Figure 1J-K). Consistent with TEM analysis, the rescue experiment in HLE cells demonstrated that ATG16L1 overexpression significantly increased LC3 protein levels and restored autophagic dysfunction induced by CX50 deficiency (Figure 1L-M). Collectively, our results demonstrated that ATG16L1 is a crucial downstream molecule for CX50-induced autophagy in the lens.

**Figure 2:**
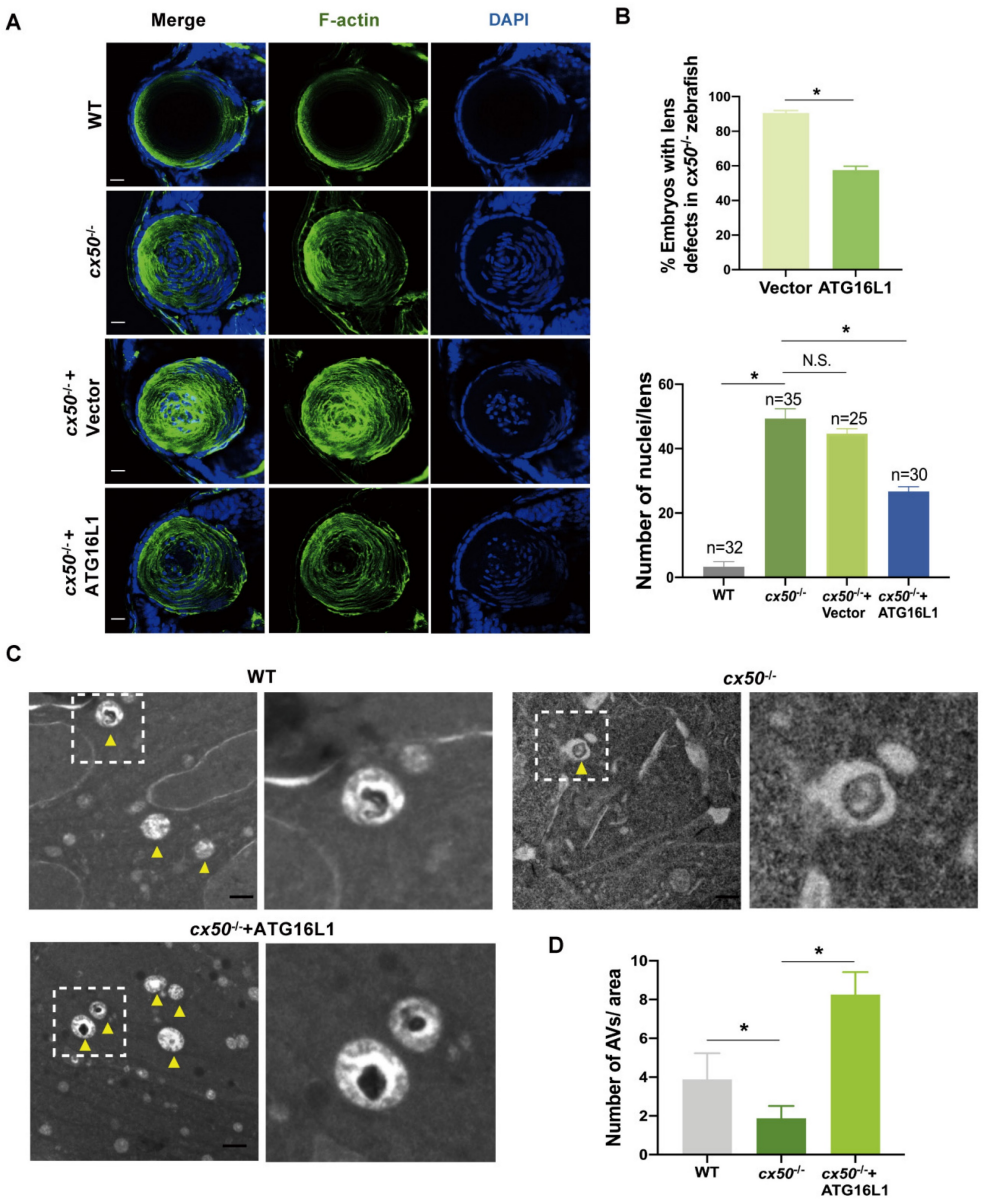
** ATG16L1 effectively relieves lens differentiation defects in *cx50*-deficient zebrafish.** (A) Representative images show the distribution of nuclei and F-actin in the lenses of 72 hpf WT, *cx50*-deficient zebrafish, and *cx50*-deficient zebrafish injected with the vector or ATG16L1 plasmid. Scale bar: 10 µm. (B) Quantification of the severity of lens defects and the number of nuclei in lenses of each group (n > 25 zebrafish for each group). Mann-Whitney test. (C) Electron micrographs of lenses in 72 hpf WT, *cx50*-deficient zebrafish, and *cx50*-deficient zebrafish injected with the ATG16L1 plasmid. Yellow arrows indicate autophagic vacuoles (AVs), scale bar: 1 µm. (D) The number of AVs per area (40 μm^2^) in each group (n ≥ 30 cells from 5 lenses). Mean ± SD, *p < 0.05. N.S. not significant (Mann-Whitney test).

Our previous work established that the *cx50*-deficient zebrafish is a typical cataract model partially caused by defective autophagy 20. We performed *in vivo* rescue experiments to further confirm the role of ATG16L1 in CX50-induced autophagy. ATG16L1 plasmids were injected into *cx50*-deficient zebrafish, and the severity of lens defects was evaluated at 72 hpf. As shown in Figures 2A and 2B, the ATG16L1 overexpression effectively relieved the lens differentiation defects in *cx50*-deficient zebrafish. The results were validated by TEM, showing increased autophagosome numbers in *cx50*-deficient zebrafish lenses with ATG16L1 overexpression (Figure 2C-D).

### ATG16L1 is mainly degraded by the ubiquitin-proteasome system (UPS)

**Figure 3:**
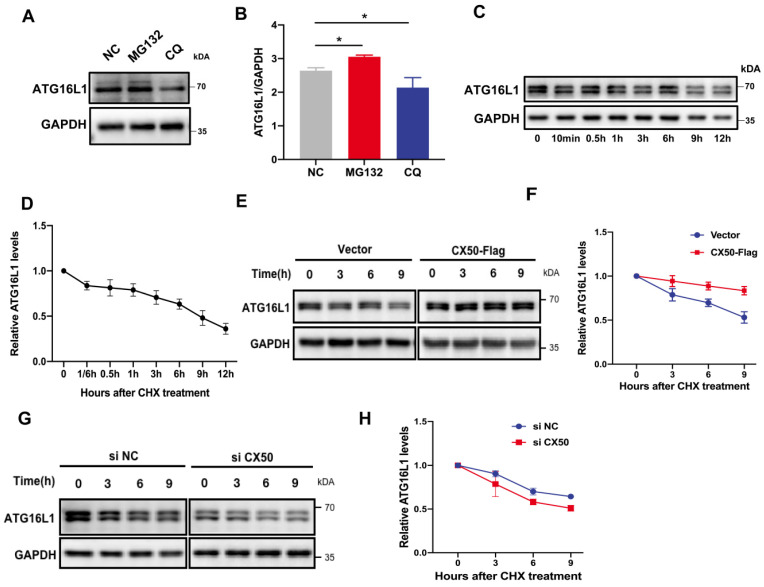
** ATG16L1 is mainly degraded by the ubiquitin-proteasome system.** (A) HLE cells were treated with 10 µM MG132 or 30 µM CQ for 6 h, and Western blotting was performed. (B) Quantification of LC3 levels, n = 3, *p < 0.05 (Mann-Whitney test). (C) HLE cells were treated with 30 µg/ml CHX for the indicated time, and the ATG16L1 expression was detected by Western blotting, n = 3. (D) ATG16L1 expression relative to GAPDH was quantified. (E) CX50-overexpressing HLE cells or (G) CX50-knockdown HLE cells were treated with 30 µM CHX for different times and (F and H) quantification of the ATG16L1 expression levels normalized to the GAPDH, n = 3, mean ± SD.

In mammalian cells, proteins undergo degradation through UPS or autophagy. Using inhibitors of these two pathways, we observed that ATG16L1 degradation was restored by the proteasomal inhibitor MG132 (Figure 3A-B), indicating that ATG16L1 turnover is mainly mediated by the UPS in HLE cells. Additionally, (Figure 3C-D), ATG16L1 in CX50-overexpressing HLE cells exhibited a longer half-life than in HLE cells under physiological conditions (Figure 3E-F), whereas CX50 knockdown HLE cells showed a shorter half-life (Figure 3G-H), indicating that CX50 may regulate ATG16L1 protein level by affecting its ubiquitin-dependent degradation.

**Figure 4:**
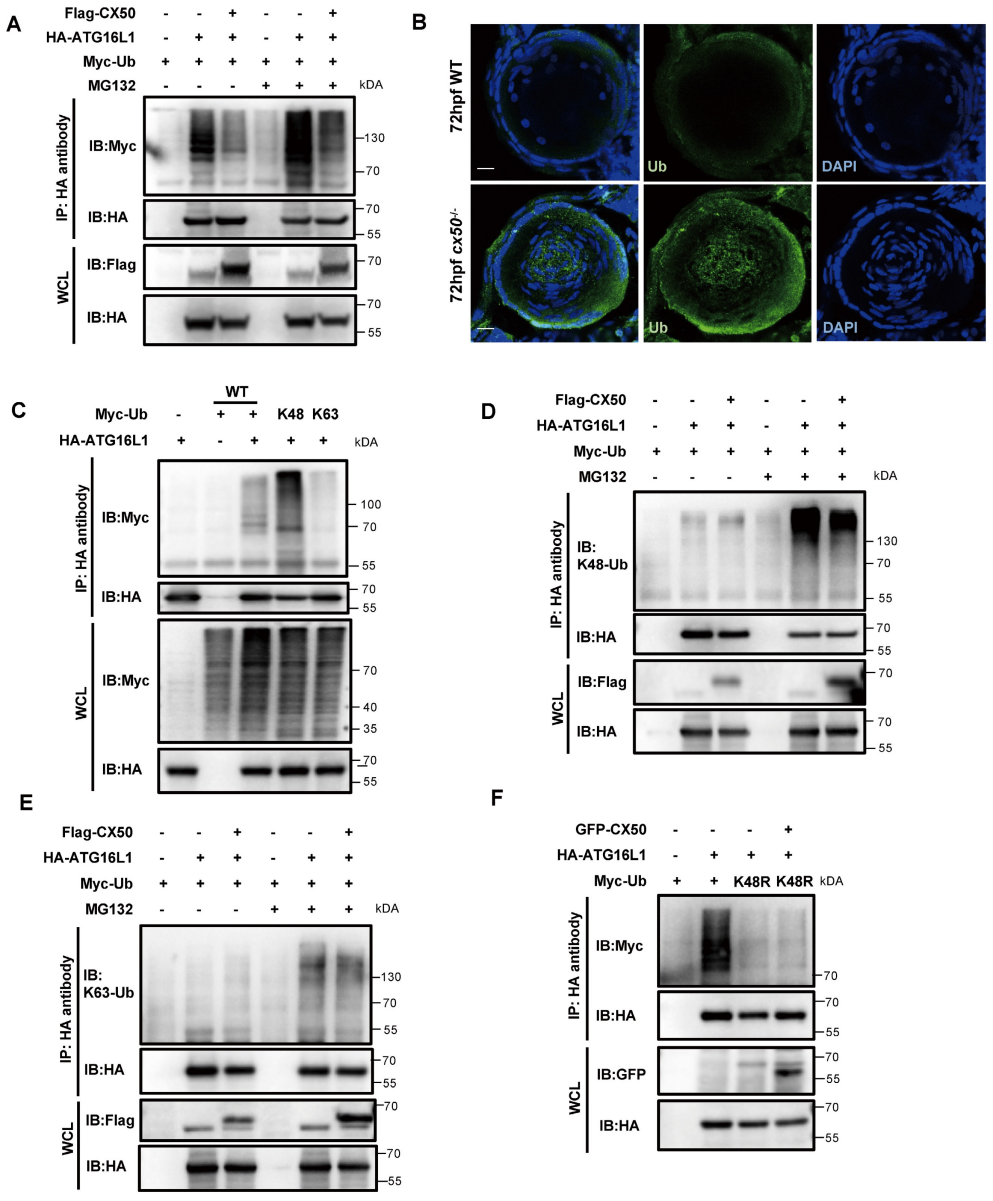
** K48-linked ubiquitination of ATG16L1 is mediated by CX50.** (A) HEK293 cells were transfected with HA-ATG16L1 or Myc-Ub with or without Flag-CX50. After 24 h transfection, IP was performed with HA affinity gels on cells with or without MG132 treatment for 6 h, and ubiquitination was analyzed using anti-Myc. WCL: whole cell lysis. (B) Representative images showing the distribution of ATG16L1 and Ub in lenses from 72 hpf WT and *cx50*-deficient zebrafish. Scale bar: 10 µm. (C-F) The HEK293 cells were transfected with indicated plasmids for 24 h, and the IP using HA affinity gels was subjected to immunoblotting using the indicated antibodies.

Ubiquitination is described as the hallmark of protein degradation via the UPS in mammalian cells. IP assays showed that CX50 overexpression markedly reduced the ubiquitination level of ATG16L1 (Figure 4A). This finding was further validated by abnormal accumulation of ubiquitin in the center of the lens in cx50-deficient zebrafish (Figure 4B). It has previously been reported that different ubiquitin chains might exert diverse biological functions 23. To determine the ubiquitination-modified ATG16L1, we co-transfected the HA-ATG16L1 and Myc-Ub (WT) with Myc-K48 or Myc-K63 plasmids in HEK293T cells (Figure 4C). Subsequently, using anti-K48 and K63-linkage polyubiquitin antibodies, we further confirmed that CX50 overexpression specifically decreased the K48-linked but not the K63-linked ubiquitination of ATG16L1 (Figure 4D-E). Additionally, IP assays employing a K48R ubiquitin mutant demonstrated attenuation of ATG16L1 ubiquitination mediated by CX50 (Figure 4F). Collectively, these results verified that CX50 inhibits ATG16L1 degradation through K48-linked ubiquitination, inducing proteasomal degradation of target proteins.

### The interaction between ATG16L1 and E3 ubiquitin ligase gigaxonin is weakened by CX50 overexpression

**Figure 5:**
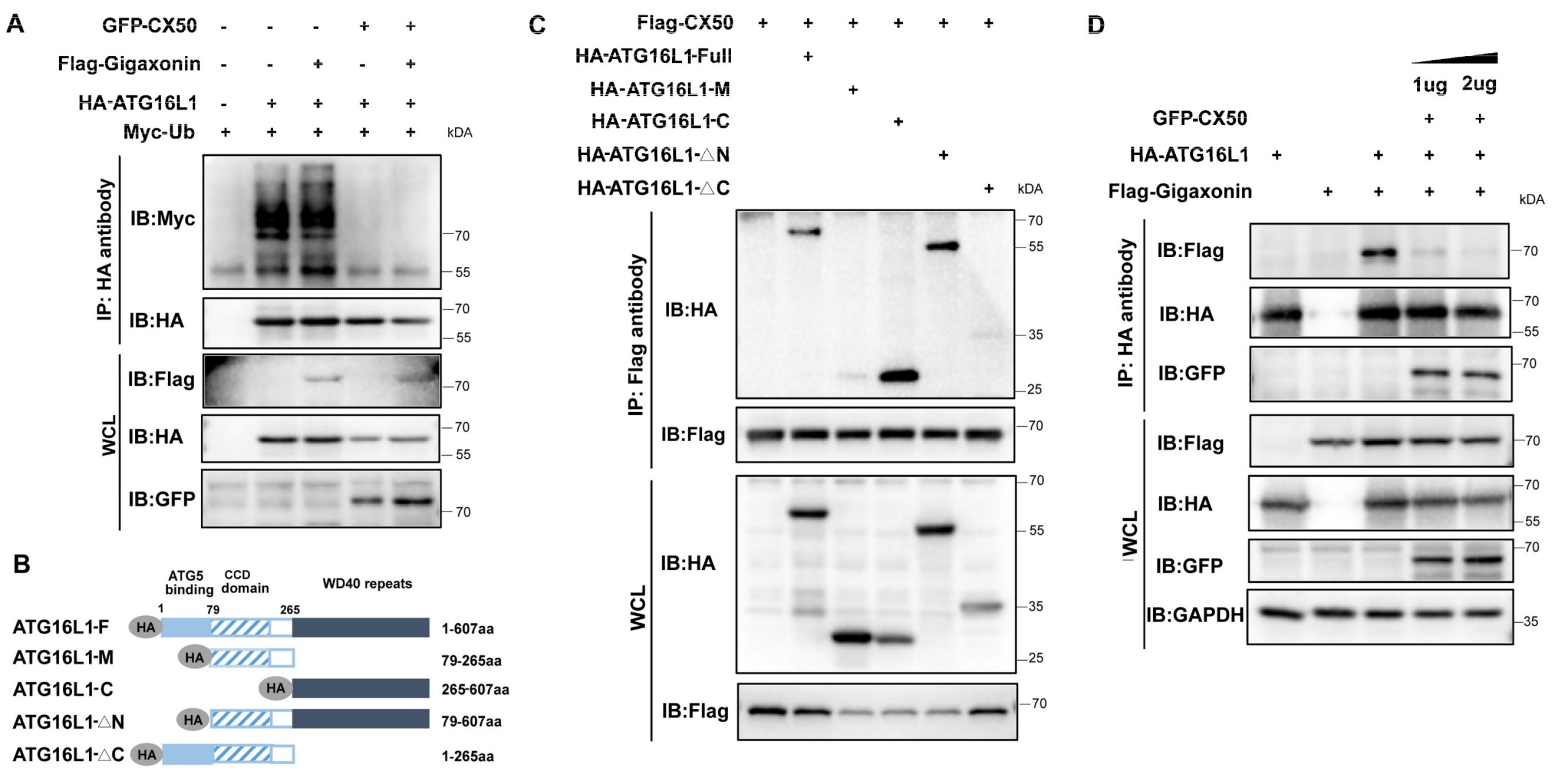
** The interaction between ATG16L1 and E3 ubiquitin ligase gigaxonin is weakened by CX50 overexpression.** (A) HEK293 cells were transfected with indicated plasmids, and IP was analyzed with HA affinity gels and Western blotting. (B) ATG16L1 deletion constructs. (C) Immunoblots of immunoprecipitates from CX50-Flag with Atg16L1-HA, ATG16L1-M, ATG16L1-C, ATG16L1-△N, and ATG16L1-△C in HEK293T cells. WCL: whole cell lysis. (D) HEK293 cells were transfected with CX50-GFP, ATG16L1-HA, or gigaxonin-Flag for 24 h. ATG16L1-HA was then immunoprecipitated by anti-HA, and the associated gigaxonin and ATG16L1 were analyzed by Western blotting.

Gigaxonin has been reported to be the only E3 ligase of ATG16L1 19. Co-IP assays revealed that overexpressed gigaxonin increased ATG16L1 ubiquitination (Figure 5A). However, CX50 overexpression significantly inhibited this process in vitro (Figure 5A). We investigated the ATG16L1-binding domains of CX50 to uncover the potential regulatory mechanism. ATG16L1 comprises three main domains: the N-terminal domain, the coiled-coil domain (CCD), and the C-terminal WD40 domain (WDD). We co-expressed Flag-CX50 with HA-tagged full-length ATG16L1 or one of four ATG16L1 deletion constructs (ATG16L1-M, ATG16L1-C, ATG16L1-△N and ATG16L1-△C) in 293T cells and performed CoIP assays (Figure 5B). As shown in Figure 5C, the ATG16L1-C and ATG16L1-△N, containing the WDD domains, exhibited affinity for CX50 similar to the full-length ATG16L1 protein, suggesting CX50 as a WDD-interacting protein. Since gigaxonin was reported to interact with ATG16L1 via the WD40 domain 19, we explored the potential relationship between CX50 and gigaxonin. The results revealed that the dissociation of gigaxonin from ATG16L1 was proportional to the amount of CX50 added (Figure 5D). These data suggested that CX50 weakens the interaction between gigaxonin and ATG16L1, thereby inhibiting gigaxonin-mediated ubiquitination of ATG16L1.

### Identification of riboflavin as a potential ATG16L1 ubiquitination regulator

**Figure 6:**
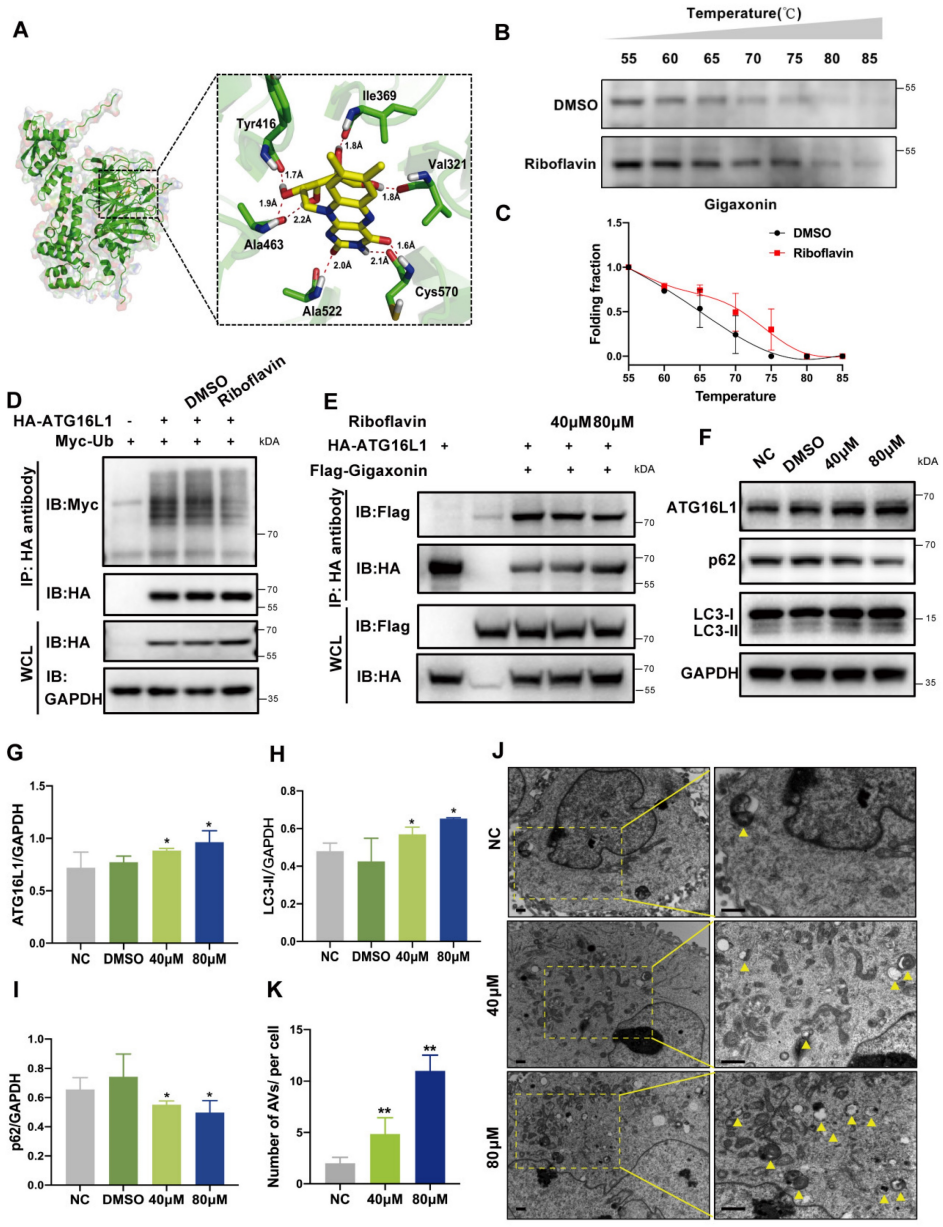
** Identification of Riboflavin, a potential ATG16L1 ubiquitination regulator.** (A) Modeling of riboflavin docking superimposed on gigaxonin, with the interacting gigaxonin amino acids represented by green sticks. (B) Cellular thermal shift assay of gigaxonin with riboflavin. (C) gigaxonin melting curves, n = 3. (D-E) HEK293 cells were transfected with indicated plasmids for 24 h; then, the cells were treated with DMSO or riboflavin for another 24 h. Western blots of immunoprecipitates with HA affinity gels. (F) Western blots of ATG16L1, LC3, and p62 protein levels in HLE cells treated with DMSO or riboflavin (40 µM and 80 µM), n = 3. (G-I) Quantification of ATG16L1, LC3, and p62 levels, n = 3 (Mann-Whitney test). (J-K) Electron micrographs of HLE cells and quantification of AVs per cell. Yellow arrows indicate autophagic vacuoles. ≥ 8 cells per experiment, scale bar: 1 µm. Mean ± SD, *p < 0.05, **p < 0.01(Mann-Whitney test).

E3 ubiquitin ligase is widely believed to be a viable drug target for various diseases. We explored its potential applications in cataract treatments by employing the crystal structure of E3 ligase gigaxonin and conducting the docking screening of a chemical library. We found a potential interaction between gigaxonin and riboflavin. As shown in Figure 6A, riboflavin binds to gigaxonin via amino acids ALA522, CYS570, TYR416, ALA463, VAL321, and ILE369. Subsequently, we confirmed the direct binding of riboflavin to gigaxonin using the cellular thermal shift assay (CETSA) in HLE cells. Figures 6B-C show that adding 80 µM riboflavin increased the stability of gigaxonin. These results suggested that riboflavin directly targets gigaxonin.

Co-IP assays showed that riboflavin could attenuate ATG16L1 ubiquitination (Figure 6D). Furthermore, riboflavin caused the dissociation of gigaxonin from ATG16L1 in a dose-dependent manner (Figure 6E). When the ATG16L1 protein expression and autophagy activity were examined with different concentrations of riboflavin, a dose-dependent increase in ATG16L1 and LC3 protein levels and a decrease in the p62 level were observed (Figure 6F-I). Consistently, riboflavin treatment led to a significant increase of autophagosome formation in HLE cells (Figure 6J-K). These data suggested that riboflavin can attenuate ATG16L1 ubiquitination, thus increasing the ATG16L1 protein level to promote autophagy in HLE cells.

### Riboflavin alleviates lens opacity in autophagy-related cataract models

**Figure 7:**
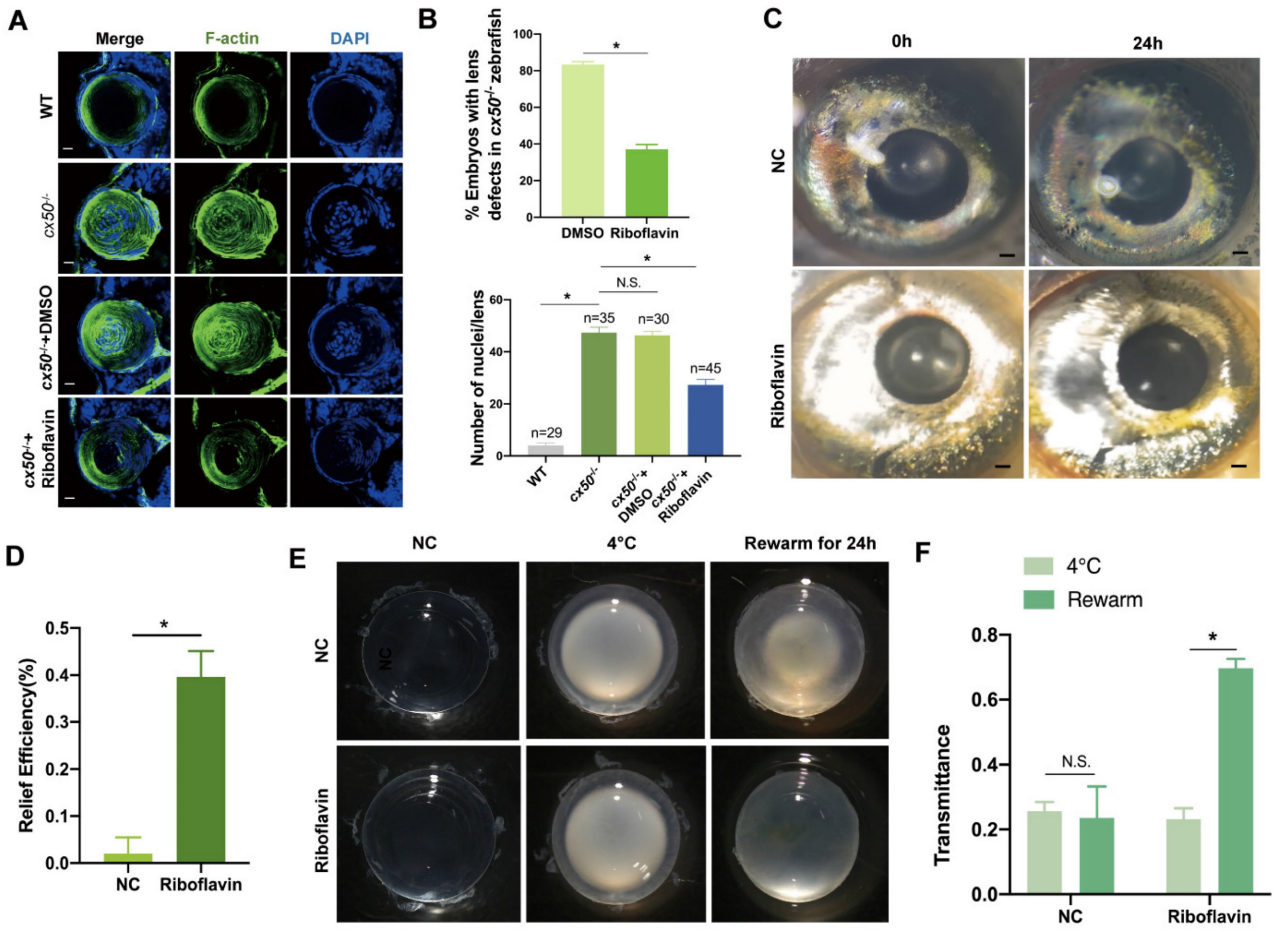
** Riboflavin alleviates lens opacity in autophagy-related cataract models.** (A) Representative images show the distribution of nuclei and F-actin in the lens of 72 hpf WT, *cx50*-deficient zebrafish, and *cx50*-deficient zebrafish treated with DMSO or riboflavin (80 µM). Scale bar: 10 µm. (B) Quantification of the severity of lens defects and the number of nuclei in lenses of each group (n > 25 zebrafish for each group) (Mann-Whitney test). (C) Representative images show that riboflavin alleviates lens opacity in the H_2_O_2_-induced cataract zebrafish model (n > 6 for each group). (D) Quantification of relief efficiency (Mann-Whitney test). (E) Representative images show that riboflavin could relieve cataract phenotype in cold-induced cataract rat models. (F) Quantification of transmittance (n > 5 for each group). Mean ± SD, *p < 0.05. N.S. not significant (Mann-Whitney test).

Based on the promising *in vitro* results of riboflavin observed in cells, we investigated its efficacy *in vivo* in autophagy-related cataract models. First, we observed that riboflavin treatment prompted the denucleation and loss of the cytoskeleton and relieved lens opacity in *cx50*-deficient zebrafish (Figure 7A-B). Intriguingly, the cataract in the H_2_O_2_-induced zebrafish model was also alleviated after treatment with riboflavin (Figure 7C-D). Similarly, the chemical rescue effect was also observed in the cold-induced cataract rat models. Compared with the control group, transmittance was effectively improved following riboflavin treatment (Figure 7E-F). Collectively, these results suggested that riboflavin treatment relieves symptoms in multiple autophagy-related cataract models.

## Discussion

The lens, a transparent and avascular tissue, relies on programmed organelle degradation for development and maintenance, involving autophagy and other complex mechanisms. A significant finding of our study is that excessive degradation of ATG16L1 through the UPS, catalyzed by the E3 ubiquitin ligase gigaxonin, disrupts autophagy in the* cx50*-deficient cataract model. Additionally, we discovered that riboflavin could regulate ATG16L1 ubiquitination, promoting autophagy and reducing lens opacity in various autophagy-related cataract models, including *cx50*-deficient zebrafish, H_2_O_2_-induced cataract zebrafish, and cold-induced cataract rat models. These findings suggested ATG16L1 ubiquitination as a potential target for cataract treatment.

Autophagy plays a pivotal role in maintaining lens homeostasis 24. It helps preserve transparency by eliminating organelles and acts as a protective mechanism against factors like ultraviolet radiation 25, 26. Disruption of autophagy through the knockdown of core autophagy proteins or regulators, such as Atg5 and TBC1D20, has been linked to cataract development 10, 27. Studies have shown that the absence of ATG5 in the lens leads to age-related cataracts due to the accumulation of damaged proteins and insoluble crystallins 27. Moreover, oxidative stress triggers abnormal autophagic responses during the aging process of human lens epithelial cells 28, 29.

Our previous study demonstrated organelle degradation defects due to defective autophagy in the *cx50* mutant zebrafish cataract model 20. This study further validated impaired autophagic flux and autophagosome formation in HLE cells with CX50 deficiency. We provided *in vitro* and *in vivo* evidence supporting ATG16L1 as a downstream target for CX50 in regulating autophagy in the lens. Our finding was consistent with a previous report showing that connexins interact with specific ATGs in the plasma membrane, influencing basal autophagy function 30. Additionally, CX43, another protein extensively expressed in the lens, was found to interact with ATG16L1 C-terminus, acting as a negative autophagy regulator 30.

Notably, our *in vivo* experiments demonstrated that injecting the ATG16L1 plasmid increased autophagosome numbers, improved organelle degradation, and alleviated cataract symptoms in *cx50*-deficient zebrafish. These findings underlined the importance of ATG16L1 in cataract formation. ATG16L1, a key autophagy-related protein, plays significant roles in various diseases, from inflammatory bowel diseases, like Crohn's disease, to neurodegenerative diseases, such as Alzheimer's disease 31, 32. Additionally, targeting the ATG16L1 axis was effective in modulating autophagy and overcoming drug resistance in cancer 33. Our present study provides direct evidence of ATG16L1's involvement in cataract formation.

Ubiquitination, a crucial post-translational modification, regulates the activities of autophagy-related proteins (e.g., Beclin1, ULK1, and p62) and key regulators (e.g., AKT, mTORC1, and AMPK) that control autophagic processes 34-39. Ubiquitination involves a sequence of actions, including E1, E2, and E3 ubiquitin ligases, with E3 ligases determining substrate specificity 23, 40. Recent studies have confirmed that gigaxonin E3 ligase regulates ATG16L1 ubiquitination, affecting autophagy in neurodegenerative diseases 19. Our study found that ATG16L1 turnover predominantly occurs through the UPS process in HLE cells. The Co-IP assay showed that CX50 binds to ATG16L1, causing its dissociation from gigaxonin, reducing ATG16L1 K48 ubiquitination, and preserving its stability in the lens, thus identifying a potential mechanism for autophagy regulation in lens homeostasis.

E3 ubiquitin ligases are recognized as potential multi-drug targets for various diseases 41. We employed the virtual screening technology, commonly used in other diseases, like liver cancer and colorectal cancer 42, to identify a potential interaction between gigaxonin and riboflavin, a water-soluble B vitamin 43. Strikingly, our results confirmed that riboflavin could increase ATG16L1 levels by reducing its ubiquitination, promoting autophagy, and rescuing the cataract phenotype in the *cx50*-deficient zebrafish model. Also, we validated the effectiveness of riboflavin treatment in two other cataract models associated with autophagy: the H2O2-induced cataract zebrafish model, characterized by over-activated oxidative stress-induced autophagy disturbance 29, 44, 45 and the cold-induced cataract rat model, where autophagy acts as a protective mechanism during hibernation 46, 47. Our results showed that riboflavin effectively relieved lens opacity in all models tested. Notably, riboflavin deficiency has been reported in 80% of cataract patients 43. As a safe drug with no known toxicity, riboflavin has been widely used in antioxidant and anti-inflammatory treatments for conditions like keratoconus, cheilitis, and sepsis. Our study suggests riboflavin as a potential therapeutic alternative for cataract treatment.

In summary, it is well-established that autophagy plays a role in cataract formation. Our results emphasize the critical role of ATG16L1 ubiquitination in modulating autophagy during cataract development, providing evidence for the intricate relationship between UPS and the autophagy pathway. Our study underscores the potential for drug design targeting E3 ubiquitin ligases to regulate autophagy, advancing the treatment of diseases associated with autophagy.

## Supplementary Material

Supplementary figure, table, and information.

## Abbreviations

ATG: Autophagy-related gene
CX50: connexin 50
CQ: chloroquine
CHX: cycloheximide
HLE cells: Human Lens Epithelial cells
Hpf: hours post-fertilization

## References

[1] (2021). Causes of blindness and vision impairment in 2020 and trends over 30 years, and prevalence of avoidable blindness in relation to VISION 2020: the right to sight: an analysis for the global burden of disease study. Lancet Glob Health.

[2] Chen X, Xu J, Chen X, Yao K (2021). Cataract: Advances in surgery and whether surgery remains the only treatment in future. Adv Ophthalmol Pract Res.

[3] Levine B, Kroemer G (2019). Biological functions of autophagy genes: a disease perspective. Cell.

[4] Noda NN, Inagaki F (2015). Mechanisms of autophagy. Annu Rev Biophys.

[5] Li H, Gao L, Du J, Ma T, Li W, Ye Z (2023). Impacts of autophagy on the formation of organelle-free zone during the lens development. Mol Biol Rep.

[6] Brennan L, Costello MJ, Hejtmancik JF, Menko AS, Riazuddin SA, Shiels A (2023). Autophagy requirements for eye lens differentiation and transparency. Cells.

[7] Khan SY, Ali M, Kabir F, Na CH, Delannoy M, Ma Y (2022). The role of FYCO1-dependent autophagy in lens fiber cell differentiation. Autophagy.

[8] Sagona AP, Nezis IP, Stenmark H (2014). Association of CHMP4B and autophagy with micronuclei: implications for cataract formation. Biomed Res Int.

[9] Byrne S, Jansen L, JM UK-I, Siddiqui A, Lidov HG, Bodi I (2016). EPG5-related Vici syndrome: a paradigm of neurodevelopmental disorders with defective autophagy. Brain.

[10] Sidjanin DJ, Park AK, Ronchetti A, Martins J, Jackson WT (2016). TBC1D20 mediates autophagy as a key regulator of autophagosome maturation. Autophagy.

[11] Wignes JA, Goldman JW, Weihl CC, Bartley MG, Andley UP (2013). p62 expression and autophagy in αB-crystallin R120G mutant knock-in mouse model of hereditary cataract. Exp Eye Res.

[12] Makley LN, McMenimen KA, DeVree BT, Goldman JW, McGlasson BN, Rajagopal P (2015). Pharmacological chaperone for α-crystallin partially restores transparency in cataract models. Science.

[13] Tu C, Li H, Liu X, Wang Y, Li W, Meng L (2021). TDRD7 participates in lens development and spermiogenesis by mediating autophagosome maturation. Autophagy.

[14] Hashemi H, Pakzad R, Yekta A, Aghamirsalim M, Pakbin M, Ramin S (2020). Global and regional prevalence of age-related cataract: a comprehensive systematic review and meta-analysis. Eye (Lond).

[15] Mizushima N, Yoshimori T, Ohsumi Y (2011). The role of Atg proteins in autophagosome formation. Annu Rev Cell Dev Biol.

[16] Itakura E, Mizushima N (2010). Characterization of autophagosome formation site by a hierarchical analysis of mammalian Atg proteins. Autophagy.

[17] Fujita N, Itoh T, Omori H, Fukuda M, Noda T, Yoshimori T (2008). The Atg16L complex specifies the site of LC3 lipidation for membrane biogenesis in autophagy. Mol Biol Cell.

[18] Hamaoui D, Subtil A (2022). ATG16L1 functions in cell homeostasis beyond autophagy. FEBS J.

[19] Scrivo A, Codogno P, Bomont P (2019). Gigaxonin E3 ligase governs ATG16L1 turnover to control autophagosome production. Nat Commun.

[20] Ping X, Liang J, Shi K, Bao J, Wu J, Yu X (2021). Rapamycin relieves the cataract caused by ablation of Gja8b through stimulating autophagy in zebrafish. Autophagy.

[21] Zhang M, Luo J, Chen X, Chen Y, Li P, Zhang G (2022). Identification and integrated analysis of the miRNA-mRNA regulatory network in lens from an H(2)O(2)-induced zebrafish cataract model. Curr Eye Res.

[22] Cui Y, Yang H, Shi S, Ping X, Zheng S, Tang X (2022). TP53INP2 contributes to TGF-beta2-induced autophagy during the epithelial-mesenchymal transition in posterior capsular opacification development. Cells.

[23] van Wijk SJ, Fulda S, Dikic I, Heilemann M (2019). Visualizing ubiquitination in mammalian cells. EMBO Rep.

[24] Klionsky DJ (2007). Autophagy: from phenomenology to molecular understanding in less than a decade. Nat Rev Mol Cell Biol.

[25] Fernandez-Albarral JA, de Julian-Lopez E, Soler-Dominguez C, de Hoz R, Lopez-Cuenca I, Salobrar-Garcia E (2021). The role of autophagy in eye diseases. Life (Basel).

[26] Morishita H, Mizushima N (2016). Autophagy in the lens. Exp Eye Res.

[27] Morishita H, Eguchi S, Kimura H, Sasaki J, Sakamaki Y, Robinson ML (2013). Deletion of autophagy-related 5 (Atg5) and Pik3c3 genes in the lens causes cataract independent of programmed organelle degradation. J Biol Chem.

[28] Zhou J, Yao K, Zhang Y, Chen G, Lai K, Yin H (2016). Thioredoxin binding protein-2 regulates autophagy of human lens epithelial cells under oxidative stress via inhibition of akt phosphorylation. Oxid Med Cell Longev.

[29] Li T, Huang Y, Zhou W, Yan Q (2020). Let-7c-3p regulates autophagy under oxidative stress by targeting ATG3 in lens epithelial cells. Biomed Res Int.

[30] Bejarano E, Yuste A, Patel B, Stout RF Jr, Spray DC, Cuervo AM (2014). Connexins modulate autophagosome biogenesis. Nat Cell Biol.

[31] Diamanti MA, Gupta J, Bennecke M, De Oliveira T, Ramakrishnan M, Braczynski AK (2017). IKKalpha controls ATG16L1 degradation to prevent ER stress during inflammation. J Exp Med.

[32] Wu X, Fleming A, Ricketts T, Pavel M, Virgin H, Menzies FM (2016). Autophagy regulates Notch degradation and modulates stem cell development and neurogenesis. Nat Commun.

[33] Zhang K, Chen J, Zhou H, Chen Y, Zhi Y, Zhang B (2018). PU.1/microRNA-142-3p targets ATG5/ATG16L1 to inactivate autophagy and sensitize hepatocellular carcinoma cells to sorafenib. Cell Death Dis.

[34] Shen Y, Liu WW, Zhang X, Shi JG, Jiang S, Zheng L (2020). TRAF3 promotes ROS production and pyroptosis by targeting ULK1 ubiquitination in macrophages. FASEB J.

[35] Li X, Yang KB, Chen W, Mai J, Wu XQ, Sun T (2021). CUL3 (cullin 3)-mediated ubiquitination and degradation of BECN1 (beclin 1) inhibit autophagy and promote tumor progression. Autophagy.

[36] Rogov V, Dotsch V, Johansen T, Kirkin V (2014). Interactions between autophagy receptors and ubiquitin-like proteins form the molecular basis for selective autophagy. Mol Cell.

[37] Li Y, Li S, Wu H (2022). Ubiquitination-proteasome system (UPS) and autophagy two main protein degradation machineries in response to cell stress. Cells.

[38] Yang WL, Wang J, Chan CH, Lee SW, Campos AD, Lamothe B (2009). The E3 ligase TRAF6 regulates Akt ubiquitination and activation. Science.

[39] Jiang P, Ren L, Zhi L, Yu Z, Lv F, Xu F (2021). Negative regulation of AMPK signaling by high glucose via E3 ubiquitin ligase MG53. Mol Cell.

[40] Popovic D, Vucic D, Dikic I (2014). Ubiquitination in disease pathogenesis and treatment. Nat Med.

[41] Saravanan KM, Kannan M, Meera P, Bharathkumar N, Anand T (2022). E3 ligases: a potential multi-drug target for different types of cancers and neurological disorders. Future Med Chem.

[42] Wang Z, Wang M, Zhang M, Xu K, Zhang X, Xie Y (2022). High-affinity SOAT1 ligands remodeled cholesterol metabolism program to inhibit tumor growth. BMC Med.

[43] Suwannasom N, Kao I, Pruss A, Georgieva R, Baumler H (2020). Riboflavin: The health benefits of a forgotten natural vitamin. Int J Mol Sci.

[44] Filomeni G, De Zio D, Cecconi F (2015). Oxidative stress and autophagy: the clash between damage and metabolic needs. Cell Death Differ.

[45] Navarro-Yepes J, Burns M, Anandhan A, Khalimonchuk O, del Razo LM, Quintanilla-Vega B (2014). Oxidative stress, redox signaling, and autophagy: cell death versus survival. Antioxid Redox Signal.

[46] Slezak J, Tribulova N, Okruhlicova L, Dhingra R, Bajaj A, Freed D (2009). Hibernating myocardium: pathophysiology, diagnosis, and treatment. Can J Physiol Pharmacol.

[47] Chang H, Peng X, Yan X, Zhang J, Xu S, Wang H (2020). Autophagy and Akt-mTOR signaling display periodic oscillations during torpor-arousal cycles in oxidative skeletal muscle of daurian ground squirrels (spermophilus dauricus). J Comp Physiol B.

