# Supplementary material for: Lens autophagy protein ATG16L1: a potential target for cataract treatment
Source: Theranostics. 2024 Jul 1;14(10):3984–96. doi: 10.7150/thno.93864 (PMC11234268; doi:10.7150/thno.93864)
Supplement: Supplementary file 1 — Supplementary figure, table, and information. [file thnov14p3984s1.pdf]

Supplementary material

Supplementary material 1

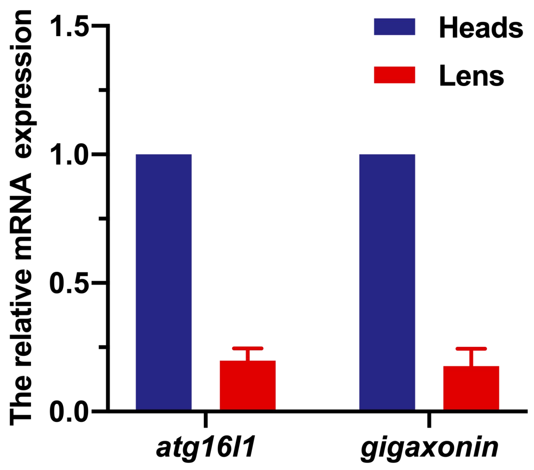

Figure S1. The mRNA expression of *atg16l1* and *gigaxonin* in zebrafish lenses and heads. n=3.

The primer sequences involved are as follows:

actb1-F: CGAGCTGTCTTCCCATCCA

actb1-R: TCACCAACGTAGCTGTCTTTCTG

atg16l1-F: CAACTCGGTGTCCTGGTCTC

atg16l1-R: GGCTCACTTTCGTTCCGTCT

gigaxonin-F: GCAGGGACAGTTTCGCATTC

gigaxonin-R: AACCACACCTCAGTCGAAGC

Supplementary material 2

Table 1. The list of chemical docking drugs related to Gigaxonin

Item	Catalog No.	Drug Name	docking score
1	HY-N0244	Theaflavin-3'-gallate	-16.420
2	HY-N6831	Xylohexaose	-16.033
3	HY-N0632	Esculentoside A	-15.961

4	HY-N0702	Tenuifolin	-15.697
5	HY-N0148	Rutin	-15.667
6	HY-B0456	Riboflavin	-15.412
7	HY-126382	Hesperidin methylchalcone	-15.406
8	HY-D0040	Calcein	-15.378
9	HY-N0619	Mulberroside A	-15.331
10	HY-N0751	Scutellarin	-15.192
11	HY-N4268	Tenuifoliside B	-15.172
12	HY-F0002	NADP (sodium salt)	-15.164
13	HY-N1407	Polygalaxanthone III	-15.161
14	HY-N0022	Isoacteoside	-15.135
15	HY-F0002A	NADP (disodium salt)	-15.094
16	HY-B0606	Diquafosol (tetrasodium)	-15.063
17	HY-108869	Fodipir	-15.013
18	HY-B0445	NAD+	-14.977
19	HY-N0703	Schaftoside	-14.928
20	HY-F0001	NADH (disodium salt)	-14.769
21	HY-N0452	Hyperoside	-14.483
22	HY-N2138	Buddlejasaponin IVb	-14.481
23	HY-N0197	Baicalin	-14.435
24	HY-N0823	Lithospermic acid	-14.380
25	HY-N2125	Parishin C	-14.370
26	HY-N1414	3',6-Disinapoylsucrose	-14.348
27	HY-N4084	Thonningianin A	-14.171
28	HY-106139	Bimosiamose	-14.166
29	HY-N0359	Cynarin	-14.116
30	HY-N2460	Aloesin	-14.096
31	HY-N2094	Genipin 1-尾-D-gentiobioside	-13.955
32	HY-D0183	ATP-polyamine-biotin	-13.850
33	HY-N2111	Momordicoside A	-13.845
34	HY-N2518	Agnuside	-13.759
35	HY-N0500	Mogroside III	-13.744
36	HY-F0003	NADPH (tetrasodium salt)	-13.678

37	HY-N6890	Tarasaponin VI	-13.652
38	HY-N0222	Avicularin	-13.608
39	HY-18963	Lavendustin A	-13.491
40	HY-N0020	Echinacoside	-13.420
41	HY-P0322	GRGDSPK	-13.304
42	HY-101964	SPI-112	-13.255
43	HY-113596	Acetyl Coenzyme A (trisodium)	-13.229
44	HY-N2497	Isoliquiritin apioside	-13.190
45	HY-N0143	Phlorizin	-13.186
46	HY-B0542	Ouabain (Octahydrate)	-13.173
47	HY-15745	PSI-7409	-13.160
48	HY-N6950	Hederacolchiside A1	-13.054
49	HY-N5012	Eurycomanone	-13.043
50	HY-N0002	(-)-Epicatechin gallate	-12.960
51	HY-N0812	Timosaponin BII	-12.958
52	HY-N0434	Astragaloside III	-12.930
53	HY-N0910	Notoginsenoside Ft1	-12.811
54	HY-N4099	Luteolin-3-O-beta-D-glucuronide	-12.803
55	HY-N4214	Isoastragaloside IV	-12.789
56	HY-N0804	Narirutin	-12.759
57	HY-N0062	Angoroside C	-12.747
58	HY-N0773	Isovitexin	-12.645
59	HY-N0311	Emodin-8-glucoside	-12.618
60	HY-N4210	Yadanziolide A	-12.522
61	HY-N0819	Raddeanin A	-12.457
62	HY-N6924	Zingibroside R1	-12.420
63	HY-N3516	Oxyresveratrol 2-O-尾-D-glucopyranoside	-12.392
64	HY-N2376	Chrysin-7-O-glucuronide	-12.378
65	HY-N0184	Glycyrrhizic acid	-12.291
66	HY-112534	GSTO-IN-2	-12.278
67	HY-N0777	Isorhamnetin-3-O-glucoside	-12.262
68	HY-N0330	Momordin Ic	-12.210
69	HY-15191	Sabutoclax	-12.094

70	HY-N0647	Silychristin	-12.068
71	HY-N6882	Genistein 8-c-glucoside	-12.055
72	HY-N3014	Bruceine D	-12.044
73	HY-N0246	Saikosaponin A	-12.005
74	HY-N6853	Mogroside I E1	-11.994
75	HY-N6814	Mogroside IIe	-11.967
76	HY-N0662	Amentoflavone	-11.851
77	HY-N0521	(-)-Gallocatechin	-11.848
78	HY-N4253	Kudinoside D	-11.843
79	HY-17638	Mizagliflozin	-11.834
80	HY-128851	Coenzyme A	-11.761
81	HY-112860	Asp-AMS	-11.697
82	HY-76225	Ammonium glycyrrhizinate	-11.686
83	HY-N0015	Astragalin	-11.678
84	HY-101854	N6-(2-Phenylethyl)adenosine	-11.642
85	HY-N0250	Saikosaponin D	-11.632
86	HY-112648	Stafib-2	-11.593
87	HY-N2118	Bilobetin	-11.580
88	HY-N0070	Solasonine	-11.557
89	HY-136303	GS-704277	-11.486
90	HY-D0193	Ponceau 4R	-11.474
91	HY-14520	Tetrahydrofolic acid	-11.400
92	HY-N0112	Dihydromyricetin	-11.377
93	HY-N6915	Mogroside II-?A	-11.371
94	HY-B0080	Folinic acid (calcium salt pentahydrate)	-11.367
95	HY-13664	Folinic acid (calcium)	-11.361
96	HY-N0319	Salvianolic acid C	-11.353
97	HY-128064	Adenosine amine congener	-11.317
98	HY-19396	Ingliforib	-11.290
99	HY-14518	Aminopterin	-11.268
100	HY-108272	Mogroside II-A2	-11.253
101	HY-12176	Aliskiren	-11.248
102	HY-108631	EB-47 (dihydrochloride)	-11.185

103	HY-N0013	Vitexin	-11.167
104	HY-P0244	Dermorphin	-11.166
105	HY-W017770	S-Adenosyl-L-methionine (disulfate tosylate)	-11.140
106	HY-15440B	Fostemsavir Tris	-11.125
107	HY-12270	T-5224	-11.122
108	HY-N2360	Hinokiflavone	-11.048
109	HY-108935	Lavendustin B	-11.006
110	HY-15005	Sofosbuvir	-11.005
111	HY-P1488	Bradykinin (1-5)	-10.986
112	HY-15568A	A-317491 (sodium salt hydrate)	-10.945
113	HY-15069	Fanapanel	-10.845
114	HY-108415	Cloprostenol sodium salt	-10.811
115	HY-107780	Cyclic-di-GMP	-10.781
116	HY-N0841	Bruceine A	-10.751
117	HY-B0596	Taltirelin	-10.747
118	HY-15229	Guadecitabine sodium	-10.709
119	HY-N2897	Dihydrokaempferol	-10.678
120	HY-13625	Ertapenem sodium	-10.661
121	HY-N4213	Anemarrhenasaponin I	-10.633
122	HY-130952	Thalidomide-O-C8-COOH	-10.626
123	HY-100207	CP21R7	-10.611
124	HY-P1187	HSDVHK-NH2	-10.589
125	HY-16637	Folic acid	-10.573
126	HY-N0136	Taxifolin	-10.559
127	HY-103460	IRL 2500	-10.559
128	HY-17556	Folinic acid	-10.530
129	HY-N5064	Bacoside A3	-10.492
130	HY-112647	Stafib-1	-10.459
131	HY-19528	SAH	-10.456
132	HY-N1447	Ganoderic acid A	-10.355
133	HY-15448	Tezacaftor	-10.341
134	HY-N0499	Cyanidin (Chloride)	-10.332
135	HY-B1300	Cefonicid (sodium)	-10.319

136	HY-P0288	[Leu5]-Enkephalin	-10.285
137	HY-138407	PD-1/PD-L1-IN 7	-10.237
138	HY-B0191	Bimatoprost	-10.195
139	HY-122524	7-Methylguanosine	-10.152
140	HY-N0355	(+)-Catechin hydrate	-10.113
141	HY-50686	Tivantinib	-10.080
142	HY-103628	PROTAC CDK9 Degrader-1	-10.078
143	HY-112646	CG-806	-10.066
144	HY-N6854	Mogroside I A1	-10.064
145	HY-120859	Zidebactam	-10.051
146	HY-N0908	Ginsenoside Rg5	-10.010
147	HY-B0360	Rebamipide	-9.987
148	HY-112862	Arg-AMS	-9.920
149	HY-N6855	Mogroside IIA1	-9.879
150	HY-D0915	Brilliant Blue FCF	-9.878
151	HY-19436	Solabegron	-9.874
152	HY-120635	BMS-1001 (hydrochloride)	-9.874
153	HY-111553	TAS0728	-9.834
154	HY-17383	Levomefolate (calcium)	-9.834
155	HY-N0657	Pinoresinol Diglucoside	-9.806
156	HY-18776	A2AR-agonist-1	-9.765
157	HY-14519	Methotrexate	-9.765
158	HY-12489	Ponceau S	-9.761
159	HY-13667	Levoleucovorin (Calcium)	-9.748
160	HY-120085	PFE-360	-9.709
161	HY-N0840	Bruceantin	-9.656
162	HY-125286	AB-680	-9.655
163	HY-129603	SI-109	-9.644
164	HY-B1297	Ceforanide	-9.644
165	HY-19332	Kifunensine	-9.638
166	HY-14301	Olodaterol	-9.628
167	HY-B1493	Bentiromide	-9.626
168	HY-16025	EOC317	-9.613

169	HY-N8537	Enfumafungin	-9.593
170	HY-D0257	Tartrazine	-9.563
171	HY-111496	Sulfo-NHS-SS-Biotin (sodium)	-9.555
172	HY-N0706	Gracillin	-9.542
173	HY-U00116	GP531	-9.536
174	HY-P0073	Tyr-Gly-Gly-Phe-Met-OH	-9.530
175	HY-10262	BMS-536924	-9.505
176	HY-13631D	Dxd	-9.453
177	HY-15323	PRT062607 (Hydrochloride)	-9.452
178	HY-16468	Squalamine	-9.450
179	HY-103076	EZ-482	-9.426
180	HY-P0002A	Protirelin (acetate)	-9.415
181	HY-B1357	Digitoxin	-9.379
182	HY-15514	Merestinib	-9.322
183	HY-N2335	Coumestrol	-9.309
184	HY-101243	XMD16-5	-9.300
185	HY-12512	cGAMP	-9.277
186	HY-P0098	[D-Ala2]leucine-enkephalin	-9.275
187	HY-111350	FT827	-9.210
188	HY-108402 A	Cefodizime (sodium)	-9.189
189	HY-N2322	Khasianine	-9.159
190	HY-10943	GNF-7	-9.153
191	HY-15441	PF-04447943	-9.101
192	HY-114174	Fmoc-Ala-Glu-Asn-Lys-NH2	-9.041
193	HY-134673	UZH1	-9.019
194	HY-10425	A-443654	-8.998
195	HY-N1511	Ganoderic acid D	-8.992
196	HY-N6971	Cimiracemoside C	-8.988
197	HY-111365	TES-1025	-8.965
198	HY-15005A	PSI-7976	-8.950
199	HY-12980	Batefenterol	-8.933
200	HY-121144	Cefazedone	-8.896
